# Evidence of activation of vagal afferents by non-invasive vagus nerve stimulation: An electrophysiological study in healthy volunteers

**DOI:** 10.1177/0333102417717470

**Published:** 2017-06-26

**Authors:** Romain Nonis, Kevin D’Ostilio, Jean Schoenen, Delphine Magis

**Affiliations:** Headache Research Unit, University Department of Neurology CHR, Liège, Belgium

**Keywords:** Neuromodulation, evoked potential, VNS, headache, dose-response analysis

## Abstract

**Background:**

Benefits of cervical non-invasive vagus nerve stimulation (nVNS) devices have been shown in episodic cluster headache and preliminarily suggested in migraine, but direct evidence of vagus nerve activation using such devices is lacking. Vagal somatosensory evoked potentials (vSEPs) associated with vagal afferent activation have been reported for invasive vagus nerve stimulation (iVNS) and non-invasive auricular vagal stimulation. Here, we aimed to show and characterise vSEPs for cervical nVNS.

**Methods:**

vSEPs were recorded for 12 healthy volunteers who received nVNS over the cervical vagus nerve, bipolar electrode/DS7A stimulation over the inner tragus, and nVNS over the sternocleidomastoid (SCM) muscle. We measured peak-to-peak amplitudes (P1-N1), wave latencies, and N1 area under the curve.

**Results:**

P1-N1 vSEPs were observed for cervical nVNS (11/12) and auricular stimulation (9/12), with latencies similar to those described previously, whereas SCM stimulation revealed only a muscle artefact with a much longer latency. A dose-response analysis showed that cervical nVNS elicited a clear vSEP response in more than 80% of the participants using an intensity of 15 V.

**Conclusion:**

Cervical nVNS can activate vagal afferent fibres, as evidenced by the recording of far-field vSEPs similar to those seen with iVNS and non-invasive auricular stimulation.

## Introduction

Invasive vagus nerve stimulation (iVNS) is a well-known therapeutic alternative for drug-resistant epilepsy (1). Case reports have suggested that iVNS could also have beneficial effects in other disorders such as depression, Alzheimer’s disease, and primary headache (2–7). The results of these case reports were encouraging, but no large randomised controlled studies of iVNS in primary headache have been conducted to date. The use of iVNS requires surgical intervention for electrode implantation and battery placements and replacements, thereby carrying a risk of potential surgical complications (e.g. haemorrhage, lead migration, infection).

Non-invasive alternatives to iVNS have been developed to stimulate the vagus nerve transcutaneously at the cervical or auricular region using external devices for non-invasive vagus nerve stimulation (nVNS). These devices avoid the risk of surgical complications associated with iVNS. The minimal risks of nVNS offer potential benefits in the treatment of more prevalent medical diseases beyond refractory epilepsy. An nVNS device that stimulates the cervical vagus nerve in the neck is approved by the US Food and Drug Administration for the acute treatment of pain associated with episodic cluster headache (8) and has demonstrated significant preventive therapeutic effects in chronic cluster headache when used with standard of care (versus standard of care alone) (9). Preliminary evidence has also suggested potential efficacy in the treatment of migraine (10,11). The favourable safety and tolerability profile of nVNS mitigates concerns associated with the use of triptans and other pharmacologic agents, especially in certain subsets of patients (e.g. those with a medical history of cardiovascular/cerebrovascular diseases or medication overuse headache) (8–12).

The mechanism of action of vagus nerve stimulation (VNS) in the treatment of headache is probably multifactorial. The majority of afferents in the cervical portion of the vagus nerve are of visceral origin and project to the nucleus tractus solitarius (NTS), while the small population of somatic afferents project to the trigeminal nucleus caudalis (TNC) (13). The anti-nociceptive effects of iVNS have been established in rodents (14). This therapy has also demonstrated the ability to modulate both firing of spinal trigeminal nucleus neurons in response to dura mater stimulation (15) and cortical synchrony and rhythmicity through the activation of muscarinic receptors in rodents (16). Findings from another study showed that stimulating the afferent fibres of the vagus nerve in the neck (primarily Aβ and Aδ fibres) suppressed dural stimulation-induced facial allodynia in rats for more than three hours, with the nVNS-associated decrease in trigeminal pain potentially mediated by a glutamate reduction in the TNC (17).

The efficacy of cervical nVNS shown in cluster headache and suggested in migraine (8–11) has not been directly proven to be mediated through the activation of vagus nerve afferents. A functional magnetic resonance imaging (fMRI) study in healthy subjects showed that nVNS activated the NTS and several brain areas that receive vagal input and deactivated the TNC (18). In humans, iVNS is able to evoke a short-latency somatosensory nerve potential that can be recorded ipsilaterally over the scalp and has been attributed to the activation of vagus nerve sensory afferents (19–21). This far-field vagal somatosensory evoked potential (vSEP) can also be elicited after transcutaneous stimulation of the auricular branch of the vagus nerve, with three reproducible peaks (P1, N1, and P2) being identified from C4-F4 recordings and higher-intensity stimulations leading to increasing vSEP amplitudes (20,22). The vSEP latencies obtained using auricular vagal stimulation are consistent across several studies (20,22–24). In the surgical setting of direct VNS, a response consisting of four peaks (P1, N1, P2, and N2) was clearly identifiable at the scalp level (21). The late P2-N2 component of the vSEP, but not the early P1-N1 peak, disappeared after neuromuscular blockade, suggesting that the late components have a muscular origin (19,21).

Based on the above-mentioned electrophysiological studies, we aimed to determine if cervical nVNS could elicit an evoked response similar to the vSEPs previously described in the literature and to further characterise this response to better understand the mechanism of action of nVNS in the treatment of primary headaches.

## Methods

### Research participants

This investigator-initiated, single-centre study included 12 healthy volunteers (HVs; mean ± SE age, 26.9 ± 5.2 years; five females) who had no history of cervical surgery or other relevant medical procedures, no personal or familial history of headache, and no daily medication intake other than oral contraceptives. The HVs were university students or members of the hospital staff who were recruited between February and April 2015 at the Headache Research Unit, University Department of Neurology, CHR Citadelle, Liège, Belgium. The study was reviewed and approved by the local ethics committee of the Centre Hospitalier Régional de la Citadelle and was conducted in accordance with the Declaration of Helsinki. Prior to testing, all participants were provided with detailed information about the study procedures and gave their written informed consent.

### Protocol and devices

**Figure 1.**
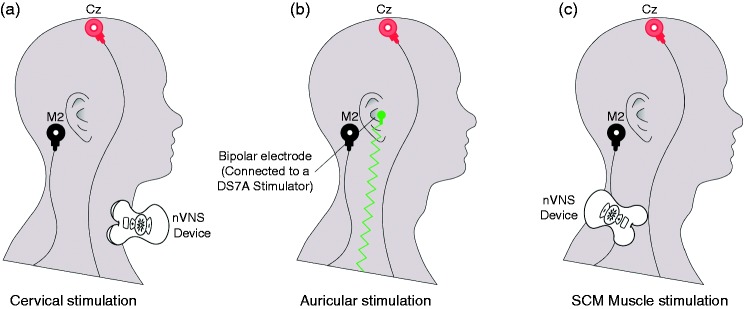
Stimulation conditions (a) at M2-Cz after nVNS over the right paramedian cervical region (first active condition); (b) at M2-Cz after bipolar stimulation over the right inner tragus (second active condition); (c) at M2-Cz after nVNS over the right SCM muscle (control condition). nVNS: non-invasive vagus nerve stimulation; SCM: sternocleidomastoid. Stimulations were performed bilaterally, with recordings performed at different electrode positions according to the side of stimulation, but only right stimulation and M2-Cz are depicted in this figure.

In each HV, vSEPs were recorded in three different conditions using bilateral stimulations on the same day in a pseudo randomised order (Figure 1): 1) the nVNS device was placed over the cervical portion of the vagus nerve (first active condition); 2) the vagus nerve afferents in the inner tragus were stimulated (second active condition); and 3) the nVNS device was placed over the sternocleidomastoid (SCM) muscle (control condition). Recordings were performed according to the side of stimulation.

In the *first active condition*, cervical nVNS was delivered using a portable CE-marked nVNS device (gammaCore®; electroCore, LLC, Basking Ridge, NJ, USA) placed over the expected location of the vagus nerve in the anterolateral cervical region (Figure 1(a)). This portable nVNS device produces a low-voltage electrical signal consisting of a 5-kHz sine wave burst lasting for 1 ms (five sine waves, each lasting 200 μs), with such bursts repeated once every 40 ms (25 Hz) for two minutes per stimulation. The vSEP registration period started at the beginning of a burst and lasted 35 ms. The stimulations were applied over the skin of the neck using two stainless steel contact surfaces covered with a small amount of highly conductive, multipurpose electrolyte gel. The electrical signal used for the nVNS stimulations ranged from 6 V to a peak of 18 V, with a maximum output current of 60 mA. The vSEPs were recorded at five different voltages (6, 9, 12, 15, and 18 V), which corresponded to pre-programmed intensities delivered by the cervical nVNS device.

In the *second active condition*, vSEPs were obtained with stimulation of vagus nerve afferents in the inner tragus (22,25). A custom-made bipolar electrode connected to an electrical stimulator (DS7A stimulator; Digitimer Ltd., Welwyn Garden City, Hertfordshire, UK) was used to stimulate the inner tragus of the ear (Figure 1(b)) because the size of this region was incompatible with the effective use of the nVNS device. The stimulation intensity was adjusted according to each participant’s individual sensitivity (mean stimulation intensity, ≈8 mA). Fifty stimulations were delivered at a frequency of 2 Hz and a pulse duration of 500 ms over the medial region of the tragus close to the entry of the acoustic meatus.

A *control condition* was used to distinguish vagal nerve potentials from muscular artefacts by positioning the cervical nVNS device over the SCM muscle in the posterolateral part of the neck using a stimulation intensity of 9 V (Figure 1(c)).

### vSEP recordings and analyses

Needle electrodes were placed on the scalp at M1/2, Cz, C3/4, and F3/4 according to the International EEG 10-20 system. The vSEPs were recorded ipsilateral to the side of stimulation, and the M1/2-Cz and C3/4-F3/4 electrode configurations were evaluated. A ground electrode was placed over the wrist. The vSEPs were averaged offline using CED 1401 and 1902 devices (Signal 4.11 Software; Cambridge Electronic Design, Cambridge, UK).

The vSEP peaks appearing up to 10 ms (P1, N1) and latencies were identified as described previously (20,21). Peak-to-peak amplitudes (P1-N1) and wave latencies were measured for each cervical nVNS stimulation intensity and for auricular vagal stimulation. The P2 and N2 peaks appearing after 10 ms, latencies, and amplitudes were also measured to show muscular components.

For the dose-response analysis, after an initial DC subtraction, the evoked responses were imported into EEGLAB (MATLAB R2016a; MathWorks, Inc., Natick, MA, USA) for processing (26). An automatic artefact epoch rejection function from EEGLAB was used to remove epochs exceeding two standard deviations (SDs) from the mean channel limit. The N1 area under the curve (AUC) in M1/2-Cz was extracted from each recording where a response was clearly identifiable. The extracted values were used to construct a dose-response curve, with the dose corresponding to the logarithm of the stimulation intensity and response corresponding to the AUC value for the evoked responses. Curve-fitting analyses were performed afterwards using the mean AUC value for each stimulation intensity.

For latency and amplitude analyses, the distribution of variables was first analysed using a Shapiro-Wilk normality test. Non-parametric tests were performed in the case of non-normal distributions (i.e. the Mann-Whitney test). Latencies and amplitudes were averages of the right and left stimulations. Percentages of participants who had a clearly identifiable response to stimulation were compared among all stimulation intensities using a chi-squared goodness-of-fit test. A *p* value of < 0.05 was considered significant for all statistical evaluations. Statistical analyses were performed and graphs were developed using GraphPad Prism Windows version 6.00 (GraphPad Software, Inc., La Jolla, CA, USA).

## Results

Results are summarised in Table 1.

**Table 1.** Latencies and amplitudes for each treatment condition and electrode configuration.

Outcome^a^	Electrode configuration^b^	Cervical nVNS										Auricular vagal stimulation		SCM muscle stimulation	
		6 V		9 V		12 V		15 V		18 V		≈8 mA		9 V	
Mean	SD	Mean	SD	Mean	SD	Mean	SD	Mean	SD	Mean	SD	Mean	SD
P1 latency (ms)	M1/2-Cz	2.47	0.33	2.09	0.84	1.80	0.52	1.76	0.54	1.58	0.48	2.18	0.84	–	–
C3/4-F3/4	2.55	0.30	1.95	0.52	2.03	0.66	2.10	0.83	1.73	0.67	1.78	1.08	–	–
N1 latency (ms)	M1/2-Cz	5.68	1.69	5.10	0.98	5.19	0.68	5.25	0.41	4.86	0.95	3.79	0.78	–	–
C3/4-F3/4	5.91	1.75	4.68	1.09	5.33	0.81	5.06	0.52	5.14	0.89	2.74	1.57	–	–
P1-N1 amplitude (μV)	M1/2-Cz	0.21	0.25	0.64	0.48	1.18	1.04	1.26	1.22	1.87	1.25	4.31	4.79	–	–
C3/4-F3/4	0.08	0.08	0.19	0.13	0.28	0.21	0.31	0.27	0.47	0.28	0.41	0.47	–	–
P2 latency (ms)	M1/2-Cz	–	–	10.13	3.36	9.97	3.60	9.75	2.28	13.75	2.88	–	–	10.96	3.08
C3/4-F3/4	–	–	–	–	–	–	8.19	0.58	11.68	3.91	–	–	11.59	3.07
N2 latency (ms)	M1/2-Cz	–	–	19.44	0.80	20.19	3.64	19.65	3.54	24.43	3.20	–	–	20.36	3.37
C3/4-F3/4	–	–	–	–	–	–	21.17	5.58	22.09	3.73	–	–	21.15	3.29
P2-N2 amplitude (μV)	M1/2-Cz	–	–	0.81	0.03	0.70	0.44	1.15	0.79	1.49	1.52	–	–	14.88	11.16
C3/4-F3/4	–	–	–	–	–	–	0.54	0.29	0.68	0.69	–	–	8.35	5.26

nVNS: non-invasive vagus nerve stimulation; SCM: sternocleidomastoid; SD: standard deviation.

Missing values (–) represent the absence of a measurable response.

a P1 and N1 refer to peaks appearing up to 10 ms; P2 and N2 refer to peaks appearing after 10 ms.

b Latencies and amplitudes are averages of the right and left stimulations.

### vSEP peaks and latencies (P1, N1/P2, N2)

**Figure 2.**
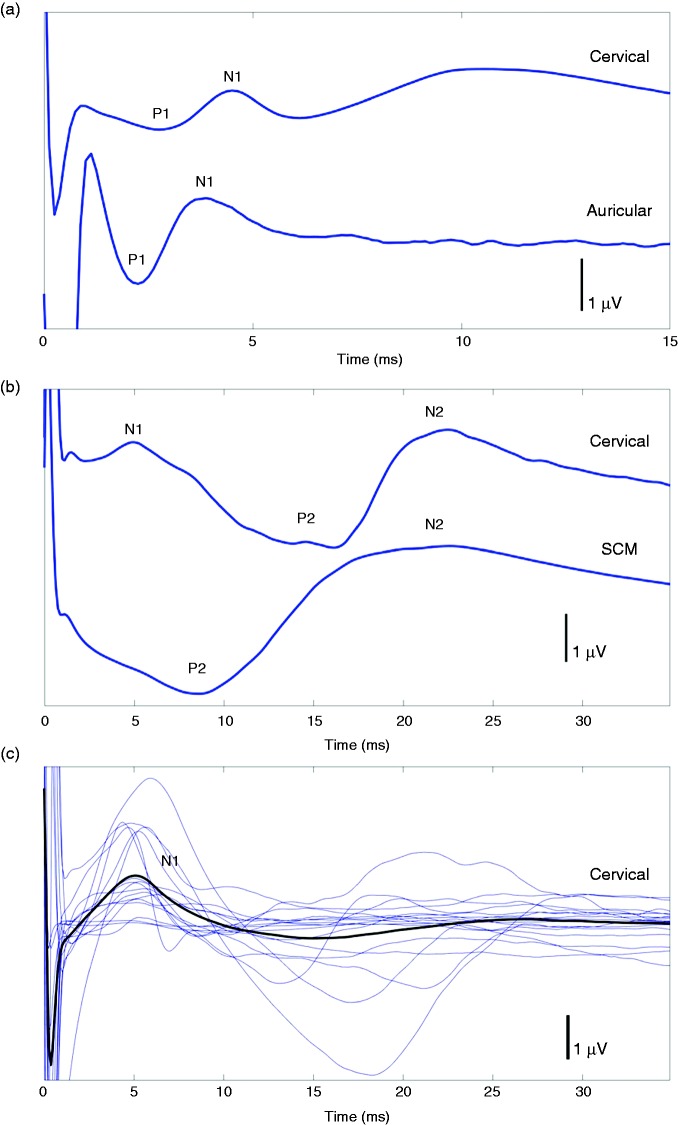
Traces in M1-Cz for (a) nVNS over the left paramedian cervical region (first active condition; top) and bipolar stimulation over the left inner tragus (second active condition; bottom) up to 15 ms in a single participant; (b) nVNS over the left paramedian cervical region (first active condition; top) and nVNS over the left SCM muscle (control condition; bottom) up to 35 ms in a single participant; and (c) nVNS over the left paramedian cervical region (first active condition) up to 35 ms in all 12 participants (blue lines) and average (black line). nVNS: non-invasive vagus nerve stimulation; SCM: sternocleidomastoid.

**Figure 3.**
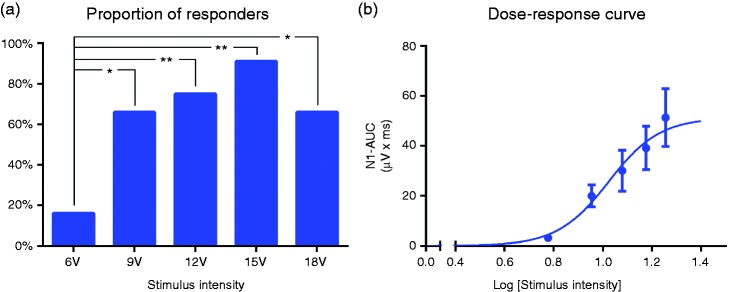
(a) Proportion of participants who had an identifiable response (at least unilaterally) using each preprogramed stimulation intensity (chi-square analysis considering all intensities); (b) logarithmic dose-response curve. AUC: area under the curve. *p < 0.05; **p < 0.01. A *responder* was defined as a participant who demonstrated at least one measurable electrophysiological signal (after left or right stimulation at any intensity) that was easily distinguishable from the baseline noise.

Cervical nVNS (first active condition) elicited two reproducible vSEP peaks (P1, N1) (Figure 2(a)) in 11 of 12 HVs on one side or both sides of neck stimulation. One of the HVs had no response to cervical nVNS throughout the session, while two participants had modest low-amplitude responses only at high stimulation intensities. The number of participants in whom a response could be evoked increased with increasing stimulation voltage, reaching a maximum of 11 participants at 15 V (Figure 3(a)). At all voltages evaluated, the tolerability of cervical nVNS was acceptable to patients. A late vSEP component (P2, N2) was also identified with cervical nVNS for nine patients in M1/2-Cz at 9, 12, 15, and 18 V and in C3/4-F3/4 at 15 and 18 V (Table 1, Figure 2(b)).

Likewise, bipolar stimulation of the inner tragus (second active condition) evoked two reproducible peaks (P1, N1) (Figure 2(a)) in nine of 12 HVs. These responses were similar to those previously described in the literature (22,24).

The SCM muscle stimulation (control condition) elicited identifiable late responses (P2, N2) (Figure 2(b)), at least unilaterally, in all 12 participants, but these responses had a much longer latency than the vSEPs elicited by cervical or auricular stimulation.

A mean N1 latency with cervical nVNS was calculated for each participant because N1 latencies of vSEPs for this active condition did not vary among different stimulation intensities (repeated measures analysis of variance (ANOVA) with Geisser-Greenhouse correction, *F*_(2.634, 18.44)_ = 1.26, *p* = 0.32). The mean ± SD N1 latencies were shorter for vSEPs elicited by auricular stimulation (second active condition) (3.79 ± 0.78 ms) than for vSEPs elicited by cervical nVNS (first active condition) (5.25 ± 0.62 ms) (*p* < 0.001). The N1 latencies for both cervical nVNS and auricular stimulation were four to five times shorter than the N2 latency for the first negative peak observed after SCM muscle stimulation (20.36 ± 3.37 ms) (both *p* < 0.001) (overall: repeated measures ANOVA with Geisser-Greenhouse correction, *F*_(1.123, 11.23)_ = 235.0; *p* < 0.010).

### P1-N1 amplitudes

The difference in mean P1-N1 vSEP amplitudes between the first and second active conditions (1.06 ± 0.78 µV and 4.31 ± 4.79 µV, respectively) was not statistically significant (*p* = 0.077).

### Dose (intensity)-response curve

The logarithmic dose-response curve (Figure 3(b)) showed a good fit over the mean AUC values for N1 responses elicited with each stimulation intensity (*R*^2 ^= 0.97). Based on this fit, the E_50_ (where 50% of the maximum response occurs) was 10.55 V (95% CI: 9.02 to 12.35).

## Discussion

Our findings suggest that cervical nVNS can elicit evoked responses that are relatively similar to those elicited by iVNS (21) and non-invasive stimulation of auricular vagal afferents in the inner tragus (22). These vSEP responses were not present when the nVNS device was moved from the vicinity of the cervical vagus nerve and was placed over the SCM muscle. Cervical nVNS was able to elicit a reproducible early P1-N1 vSEP response in 11 of 12 HVs, whereas inner tragus stimulation evoked a vSEP response in nine of 12 HVs (Figure 2). This finding might be explained by anatomical variations in the distribution of vagal afferents in the ear or differences in the number and frequency of stimuli for the two active conditions (cervical nVNS, 3000 bursts/25 Hz; auricular vagal stimulation, 50 bursts/2 Hz). Vagal afferents are absent in the tragus in up to 55% of postmortem cases but are present in 100% of cases in the cymba conchae (25), which could not be stimulated using our stimulation electrodes. Individual variations in neck anatomy could also explain the absent or minimal nVNS-induced vSEPs in two participants who had a vSEP elicited by bipolar auricular stimulation (27). Alternatively, these participants may have had a higher threshold for vagal fibre activation. Stimulations are required to pass through skin and muscular structures to reach the deeply located cervical afferents of the vagus nerve, which may explain the contraction of the platysma muscle commonly seen with cervical nVNS.

Our findings have revealed two differences between cervical and auricular vSEPs. First, the vSEP latencies were significantly shorter with auricular stimulation (3.79 ± 0.78 ms) than with cervical nVNS (5.25 ± 0.62 ms; *p* < 0.001). This difference could be related to the distance between the stimulators and the entry point of the vagus nerve into the skull. The vSEPs are thought to arise from the impedance change of the volume conductor around the vagus nerve as it enters the cranium through the jugular foramen. By changing the position of the electrode and monitoring the change in latency, Usami et al. (2013) calculated an N1 signal conduction velocity of 27 m/s at a latency of 3.3 ms (21). This velocity and latency correspond to a distance of 89.1 mm, representing the approximate distance from the electrode position on the vagus nerve to the skull base. Compared with the cervical region, the ear is probably closer to the vSEP generator thought to be localised close to the brainstem or at the skull base (21). The difference between cervical and auricular vSEP latencies could also be related to a difference in fibre populations, giving rise to different conduction velocities between the predominantly somatic auricular afferents and the predominantly visceral afferents in the cervical vagus nerve. Another difference between the vSEPs was that the P1-N1 amplitudes were slightly higher with inner tragus stimulation than with cervical nVNS (Table 1), which could also be partly related to the difference in fibre populations. One must also keep in mind that different stimulators, frequencies, and intensities were used to stimulate the vagal afferents at the auricular and cervical regions. Finally, the muscle contractions observed with cervical nVNS may partly contaminate the vSEP response and result in slight differences in wave form (Figure 2).

Vagus nerve stimulation has been considered to be a valuable therapeutic option for neurologic diseases, but its use has been limited by the need for invasive surgical procedures (28). The viability of non-invasive methods for stimulating the vagus nerve using portable devices that are more practical, convenient, and cost effective (versus iVNS) has expanded the therapeutic potential of VNS for a larger patient population and improved its accessibility for use in further studies (28,29). In our study, we observed that further increases in stimulation intensity beyond 15 V only slightly increased the responder rate and produced only a slight increase in the size of the response (Figure 3). This finding could provide clinicians with guidance suggesting that a cervical nVNS intensity of more than 15 V may not be required for the majority of patients.

The efficacy of cervical nVNS has only recently been confirmed in the treatment of cluster headache and suggested in the treatment of migraine (8–11), but its precise mode of action in these primary headaches has not been determined. The use of iVNS is known to modulate the firing of trigeminal neurons within the brainstem (15) and also to inhibit cortical synchrony and rhythmicity (15,16), while nVNS has demonstrated the ability to reduce facial allodynia and glutamate release in the TNC (17). Another study used fMRI to evaluate stimulation of the vagal afferent pathways by the nVNS device in 13 HVs (18). Activation of several vagal projections, including the NTS, was significantly greater with cervical vagal afferent stimulation by nVNS than with the control condition (18). The control stimulations involved placement of the nVNS device over the SCM muscle, consistent with the present study. The fMRI study of cervical nVNS did not include an auricular stimulation condition, but noted that the regional activity generated by the stimulation was comparable to activity reported in separate studies of non-invasive auricular vagal stimulation and iVNS (18). Like cervical nVNS, auricular stimulation increased fMRI BOLD signals in the NTS (30). Auricular stimulation also activated the locus coeruleus, with stronger nuclear activation being elicited by cymba conchae stimulation than by inner tragus stimulation (30). Our vSEP findings further support the previous fMRI evidence of vagal activation as a mechanistic explanation for the beneficial clinical effects of nVNS.

Limitations of our study include the use of surrogate markers in the absence of direct measures of vagus nerve activation. Further studies to evaluate associations between vSEPs evoked by nVNS and the clinical efficacy of this therapy are warranted. Other limitations include challenges inherent in cross-trial comparisons and controlled device studies. Cross-trial methodological differences may affect the results being compared, and effects of control conditions involving a medical device may be overstated owing to corresponding participant behaviours and perceptions (31,32). The origin of the vSEP itself has been questioned in former studies (33). The vSEPs have been suggested to correspond with an electromyographic response arising from laryngopharyngeal muscles. However, only the late P2-N2 component of the vSEP disappeared when a neuromuscular block was performed using a relaxant (19). The early P1-N1 component that we evaluated in the current study persists unchanged despite neuromuscular blockade (21). We identified a late vSEP component, as observed in the iVNS studies, similar to a late response that we reproducibly observed when applying the nVNS device over the SCM muscle (Figure 2). The P1-N1 wave was absent after stimulation over the SCM muscle, while it was identified using cervical nVNS and inner tragus stimulation, thereby favouring the involvement of nerve fibres. Finally, vSEPs elicited at auricular and neck regions differ slightly in wave form (Figure 2), possibly due to muscular contractions that cannot be avoided when using cervical nVNS.

## Conclusions

We show here for the first time that a cervical nVNS device used to treat primary headaches is able to elicit reproducible short-latency far-field sensory vSEPs similar to those elicited by iVNS and stimulation of vagus nerve branches in the ear. The amplitude of these vSEPs increased with increasing stimulation intensity and disappeared in the control condition, in which the nVNS device was positioned over the SCM muscle. Our findings suggest that vSEPs could be considered for use in therapeutic studies of nVNS and could pave the way for further trials, especially those comparing vSEP characteristics with the clinical outcomes of patients, to find accessible predictive biomarkers of nVNS efficacy.

## Article highlights

- Cervical non-invasive vagus nerve stimulation (nVNS) appears to elicit vagal somatosensory evoked potentials (vSEPs), as previously observed with invasive vagus nerve stimulation and transcutaneous auricular vagal stimulation.
- Control nVNS stimulations of the sternocleidomastoid muscle produced only longer-latency muscle artefacts.
- The vSEPs observed suggest that cervical nVNS stimulates afferent fibres of the vagus nerve.
- A dose-response analysis for cervical nVNS showed that a clear vSEP response could be elicited in more than 80% of the participants using an intensity of 15 V; cervical nVNS was well tolerated, consistent with previous studies.
- The assessment of vSEPs could lead to the development of a biomarker that is predictive of clinical responses.

## References

[1] Milby AH Halpern CH Baltuch GH Vagus nerve stimulation in the treatment of refractory epilepsy. Neurotherapeutics 2009; 6: 228–237. 19332314 10.1016/j.nurt.2009.01.010 PMC5084198

[2] Christmas D Steele JD Tolomeo S et al. Vagus nerve stimulation for chronic major depressive disorder: 12-month outcomes in highly treatment-refractory patients. J Affect Disord 2013; 150: 1221–1225. 23816447 10.1016/j.jad.2013.05.080

[3] Lenaerts ME Oommen KJ Couch JR et al. Can vagus nerve stimulation help migraine? Cephalalgia 2008; 28: 392–395. 18279429 10.1111/j.1468-2982.2008.01538.x

[4] Mauskop A Vagus nerve stimulation relieves chronic refractory migraine and cluster headaches. Cephalalgia 2005; 25: 82–86. 15658944 10.1111/j.1468-2982.2005.00611.x

[5] McGregor A Wheless J Baumgartner J et al. Right-sided vagus nerve stimulation as a treatment for refractory epilepsy in humans. Epilepsia 2005; 46: 91–96. 10.1111/j.0013-9580.2005.16404.x 15660773

[6] Merrill CA Jonsson MA Minthon L et al. Vagus nerve stimulation in patients with Alzheimer's disease: Additional follow-up results of a pilot study through 1 year. J Clin Psychiatry 2006; 67: 1171–1178. 16965193 10.4088/jcp.v67n0801

[7] Sadler RM Purdy RA Rahey S Vagal nerve stimulation aborts migraine in patient with intractable epilepsy. Cephalalgia 2002; 22: 482–484. 12133049 10.1046/j.1468-2982.2002.00387.x

[8] Silberstein SD Mechtler LL Kudrow DB et al. Non-invasive vagus nerve stimulation for the ACute Treatment of cluster headache: Findings from the randomized, double-blind, sham-controlled ACT1 study. Headache 2016; 56: 1317–1332. 27593728 10.1111/head.12896 PMC5113831

[9] Gaul C Diener HC Silver N et al. Non-invasive vagus nerve stimulation for PREVention and Acute treatment of chronic cluster headache (PREVA): A randomised controlled study. Cephalalgia 2016; 36: 534–546. 26391457 10.1177/0333102415607070 PMC4853813

[10] Grazzi L Egeo G Calhoun AH et al. Non-invasive vagus nerve stimulation (nVNS) as mini-prophylaxis for menstrual/menstrually related migraine: An open-label study. J Headache Pain 2016; 17: 91–100. 27699586 10.1186/s10194-016-0684-z PMC5047863

[11] Silberstein SD Calhoun AH Lipton RB et al. Chronic migraine headache prevention with noninvasive vagus nerve stimulation: The EVENT study. Neurology 2016; 87: 529–538. 27412146 10.1212/WNL.0000000000002918 PMC4970666

[12] Braunstein D Donnet A Pradel V et al. Triptans use and overuse: A pharmacoepidemiology study from the French health insurance system database covering 4.1 million people. Cephalalgia 2015; 35: 1172–1180. 25667299 10.1177/0333102415570497

[13] Yuan H Silberstein SD Vagus nerve and vagus nerve stimulation, a comprehensive review: Part I. Headache 2016; 56: 71–78. 26364692 10.1111/head.12647

[14] Bohotin C Scholsem M Bohotin V et al. Vagus nerve stimulation attenuates heat- and formalin-induced pain in rats. Neurosci Lett 2003; 351: 79–82. 14583386 10.1016/s0304-3940(03)00908-x

[15] Lyubashina OA Sokolov AY Panteleev SS Vagal afferent modulation of spinal trigeminal neuronal responses to dural electrical stimulation in rats. Neuroscience 2012; 222: 29–37. 22800563 10.1016/j.neuroscience.2012.07.011

[16] Nichols JA Nichols AR Smirnakis SM et al. Vagus nerve stimulation modulates cortical synchrony and excitability through the activation of muscarinic receptors. Neuroscience 2011; 189: 207–214. 21627982 10.1016/j.neuroscience.2011.05.024

[17] Oshinsky ML Murphy AL Hekierski H Jr et al. Noninvasive vagus nerve stimulation as treatment for trigeminal allodynia. Pain 2014; 155: 1037–1042. 24530613 10.1016/j.pain.2014.02.009 PMC4025928

[18] Frangos E Komisaruk BR Access to vagal projections via cutaneous electrical stimulation of the neck: fMRI evidence in healthy humans. Brain Stimul 2017; 10: 19–27. 28104084 10.1016/j.brs.2016.10.008

[19] Hammond EJ Uthman BM Reid SA et al. Electrophysiologic studies of cervical vagus nerve stimulation in humans: II. Evoked potentials. Epilepsia 1992; 33: 1021–1028. 1464258 10.1111/j.1528-1157.1992.tb01753.x

[20] Polak T Markulin F Ehlis AC et al. Far field potentials from brain stem after transcutaneous vagus nerve stimulation: Optimization of stimulation and recording parameters. J Neural Transm (Vienna) 2009; 116: 1237–1242. 19728032 10.1007/s00702-009-0282-1

[21] Usami K Kawai K Sonoo M et al. Scalp-recorded evoked potentials as a marker for afferent nerve impulse in clinical vagus nerve stimulation. Brain Stimul 2013; 6: 615–623. 23088852 10.1016/j.brs.2012.09.007

[22] Fallgatter AJ Neuhauser B Herrmann MJ et al. Far field potentials from the brain stem after transcutaneous vagus nerve stimulation. J Neural Transm (Vienna) 2003; 110: 1437–1443. 14666414 10.1007/s00702-003-0087-6

[23] Fallgatter AJ Ehlis AC Ringel TM et al. Age effect on far field potentials from the brain stem after transcutaneous vagus nerve stimulation. Int J Psychophysiol 2005; 56: 37–43. 15725488 10.1016/j.ijpsycho.2004.09.007

[24] Polak T Ehlis AC Langer JB et al. Non-invasive measurement of vagus activity in the brainstem: A methodological progress towards earlier diagnosis of dementias? J Neural Transm (Vienna) 2007; 114: 613–619. 17308983 10.1007/s00702-007-0625-8

[25] Peuker ET Filler TJ The nerve supply of the human auricle. Clin Anat 2002; 15: 35–37. 11835542 10.1002/ca.1089

[26] Delorme A and Makeig S. EEGLAB: An open source toolbox for analysis of single-trial EEG dynamics including independent component analysis. *J Neurosci Methods* 2004; 134: 9–21. 10.1016/j.jneumeth.2003.10.009 15102499

[27] Hammer N Glätzner J Feja C et al. Human vagus nerve branching in the cervical region. PLoS One 2015; 10: e0118006–e0118006. 25679804 10.1371/journal.pone.0118006 PMC4332499

[28] Magis D Neuromodulation in migraine: State of the art and perspectives. Expert Rev Med Devices 2015; 12: 329–339. 25633885 10.1586/17434440.2015.1005606

[29] Morris J Straube A Diener HC et al. Cost-effectiveness analysis of non-invasive vagus nerve stimulation for the treatment of chronic cluster headache. J Headache Pain 2016; 17: 43–51. 27102120 10.1186/s10194-016-0633-x PMC4840129

[30] Yakunina N, Kim SS and Nam EC. Optimization of transcutaneous vagus nerve stimulation using functional MRI. *Neuromodulation*. epub ahead of print 29 November 2016. doi:10.1111/ner.12541. 10.1111/ner.12541 27898202

[31] de Craen AJ Tijssen JG de Gans J et al. Placebo effect in the acute treatment of migraine: Subcutaneous placebos are better than oral placebos. J Neurol 2000; 247: 183–188. 10787112 10.1007/s004150050560

[32] Kaptchuk TJ Stason WB Davis RB et al. Sham device v inert pill: Randomised controlled trial of two placebo treatments. BMJ 2006; 332: 391–397. 16452103 10.1136/bmj.38726.603310.55 PMC1370970

[33] Leutzow B Lange J Gibb A et al. Vagal sensory evoked potentials disappear under the neuromuscular block: An experimental study. Brain Stimul 2013; 6: 812–816. 23602023 10.1016/j.brs.2013.03.005

